# The Hospital. Nursing Section

**Published:** 1902-11-29

**Authors:** 


### The Hospital.

### Nursing Section.

Contributions for this Section of "THE HOSPITAL" should be addressed to the EDITOR, "THE HOSPITAL" NURSING SECTION, 28 & 29 Southampton Street, Strand, London, W.C.

No. 844.—VOL. XXXIII. *SATURDAY, NOVEMBER* 29, 1902.

### Notes on News from the Nursing World.

### ROYALTY AND THE WEST NORFOLK HOSPITAL.

THE German Emperor was not the only person who enjoyed the shooting at Sandringham. Fifty of the pheasants were sent to the West Norfolk Hospital at Kings Lynn, and the matron and nurses thoughtfully carried the birds with their beautiful plumage round some of the wards, much to the delight of the patients. One of the men was heard to remark, "Ah, the King, he is a true English gentleman." The Queen recently visited the same hospital. The visit was quite unexpected, and her Majesty, attended by Sir Dighton Probyn, after knocking at the door, walked straight up one of the wards to the beside of an old lady from Sandringham. Some of the patients in the vicinity did not recognise her, and their excitement was great when the old lady, in reply to her visitor's kind inquiries, addressed her as "your Majesty." The Queen subsequently went through the rest of the building, and wrote her name in the visitors' book, with an expression of pleasure at the manner in which the hospital is kept. At present one of the nursing staff is attending a retainer of the Queen at Sandringham.

### REPORT OF THE DEPARTMENTAL COMMITTEE ON THE NURSING IN WORKHOUSE INFIRMARIES.

THE report of the Departmental Committee, appointed by the Local Government Board at the beginning of the year to inquire into the nursing of sick poor in workhouses, was presented to Parliament on Tuesday night, but has not yet been distributed. Probably the most interesting recommendations of the Committee are their suggestions for the establishment of "four well-defined grades" in the nursing service, namely: probationers, qualified nurses, trained nurses, and superintendent nurses. The qualifications proposed for probationers are a minimum age of 21, intelligence, good character, and health; for qualified nurses, one year's training in, and the formal certificate of, a minor training school, or, alternatively, an equivalent certificate from a non-Poor Law institution; for trained nurses, three years' training in, and the formal certificate of, a major training school, or, alternatively, an equivalent certificate from a non-Poor Law institution; and for superintendent nurses, one year's service as trained nurse or as a non-Poor Law nurse of equivalent rank, together with certificate as trained nurse, as well as midwifery qualification recognised by the Board of Midwives. Among other recommendations it is suggested that the matron, if properly trained, shall be allowed to act as trained nurse in a workhouse with not more than 60 sick beds; as superintendent nurse in a workhouse with not more than 100 sick beds; and that guardians shall be given power to appoint individual probationers without the sanction of the Local Government Board, to prescribe appropriate duties for superintendent nurses and to suspend a superintendent nurse, and shall be urged when possible to separate sick wards from the main building of the workhouse. A recommendation "that the basis of grant under the Local Government Act of 1885 shall be revised so as to enable the State to contribute more directly to the cost of the Poor Law nursing service, and that the grant, so far as nurses are concerned, shall only be paid in respect to nurses whose qualifications and appointments are in accordance with the requirements of the Local Government Board," precedes these suggestions.

### ENGLISH SISTERS ALARMED BY KAFFIRS.

AFTER the war in South Africa was over, the authorities at Pretoria came to the conclusion that barbed wires would be a sufficient protection to the nurses in their tents at night, and the guard was therefore dispensed with. But a very alarming experience followed his removal. On two occasions nurses awakened in the night to find a Kaffir standing at their bedside, and one of them was nearly frightened out of her senses. Screams brought assistance, but the intruder was not captured. The guard, however, was at once restored. Of course he ought never to have been taken away. As any of the residents could have told the authorities, special precautions should always be observed in order to prevent black men from making themselves unpleasant to white women after dark.

### A SECRET MEETING AT DUBLIN.

THE promoters of the "Coronation National Fund for Nurses in Ireland" convened a meeting on Friday last at the Royal Hospital for Incurables, Donnybrook, Dublin, matrons and nurses interested in the movement being specially invited to attend. Our representative, and, in fact, all representatives of the press, were, however, refused admission on the ground that the proceedings were "private." As no report was furnished to any of the Dublin papers, it is obvious that great precautions must have been taken to ensure the avoidance of publicity. We suspect that if a successful gathering had taken place information would have been freely furnished.

### THE DISMISSALS AT CHELTENHAM HOSPITAL.

THE letter of the honorary secretary and treasurer of Cheltenham General Hospital which appeared in our last issue has provoked rejoinders from one of the dismissed nurses and from a correspondent who seems to know what is going on behind the scenes. A singular statement in the letter of the honorary secretary has attracted their attention as well as our own. Mr. Carrington says that the nurse who was appointed sister was "an assistant nurse, not a probationer," but proceeds to add: "She will be entitled to her three years' certificate on December 4th of this year." Yet, if she has not finished her training, and has not received her certificate, how can she be otherwise than a probationer? Mr. Carrington further affirms that nine of the thirteen signatories to the petition only held a two years' certificate, but even if he be correct on this point, his defence of the management is vitiated by the fact that their training was considered good enough to qualify them for the private staff of the Cheltenham Hospital. We notice also that "One of the dismissed nurses" remarks: "Of the thirteen, four held a three years' certificate obtained at the Cheltenham Hospital, one a three years', and another a four years' certificate, obtained at other institutions." There was, therefore, no necessity to take the irregular course of selecting an uncertificated probationer. On the other hand, though there was not wanting provocation to the nurses, we cannot approve the terms of their petition. We know that it is pleaded that it was written under great excitement; but if documents of that kind are hastily composed they are apt, like hurried marriages, to be repented of at leisure. It is quite possible that a more temperately worded remonstrance would have had the desired effect.

### THE RUSH FOR THE L.O.S. CERTIFICATE.

In the report of an interview by our Commissioner with the matron of Kensington Workhouse Infirmary, which appears in another column, the significant statement is made that since the Midwives Act was passed the applications for training in midwifery have "greatly increased." In fact, at the present moment, there is no possibility of obtaining training in the wards at Kensington, and so keen is the desire to enter that the eight pupils who are taken yearly have been selected for two years. Unfortunately, it is not practicable to receive more than two pupils a quarter, but it is evident that there is an almost unlimited number of applicants from which to choose. This is all the more noteworthy, because since the Act became law the authorities at Kensington have made it a regulation not to accept any candidate for midwifery training who is not already a fully-trained nurse, and they have also imposed an age limit.

### PREPARATORY WORK IN AMERICA.

In a paper read by Miss Mary S. Gilmour, superintendent of the New York City Training School for Nurses, at the annual meeting of the American Society of Superintendents of Training Schools for Nurses in Detroit, she gave details of the preliminary course which has been adopted by her school. The classes are to be formed quarterly, and during the three months each probationer will spend her mornings in the wards and her afternoons in study under the teachers of the school. At the end of the period she will be given her junior examination in anatomy and physiology, materia medica, sanitation, and hygiene, dietetics, and practical nursing. Miss Gilmore does not approve of training schools doing their own preparatory work. She regards it as a serious tax upon the officers' time and energy, and upon the capacity, resources, and finances of the institutions which ought not to be borne by them. Her alternative is that the colleges should make provision for theoretical instruction in nursing, and that, after taking a special theoretical course at college, a woman should be able to enter a training school later for two years of practical work.

### A NURSE FOR OBER-AMMERGAU.

It may be recollected that two years ago a few friends of Ober-Ammergau started a fund to endow a bed at the Cottage Hospital in memory of the Passion Play; but it has since been found that a resident nurse living in the hospital, and going out to tend the sick in their own homes, would be a greater boon. With the consent of those who contributed to the original scheme, this proposal has been adopted. The sum of £1,000 is needed for the purpose of an endowment fund, and it is suggested that everyone who has seen and appreciated the Passion Play on any occasion since 1870-71 till the last representation in 1900 should contribute. The matter having come under the notice of the King, who with the Queen saw the play in 1871, he has sent a donation of £10 10s., with a sympathetic expression of approval, to Miss Milner, one of the promoters. Her address is Heworth Moor-house, York, and she will gladly receive subscriptions from all who desire to emulate the King's example.

### THE BLACKBURN BAZAAR.

It reflects the greatest credit on the people of Blackburn that the four days' bazaar which has just been held in the town on behalf of the Nurses' Home has yielded the quite remarkable sum of £3,582. The receipts at the nurses' stall amounted to £149 9s. 11d. Miss Chadwick, the superintendent of the Home, states that so far as she knows this is the largest amount ever made in the country on behalf of a similar cause; and such a result could certainly only have been accomplished by immense perseverance and enterprise on the part of the promoters of the bazaar, as well as by a keen appreciation on the part of the visitors of the articles on sale. About £2,000 of the sum raised will be used as an endowment fund and the remainder will go towards the liquidation of the debt on the building. It is not the least interesting feature of the bazaar that in one stall alone there were nearly a thousand contributions from working men.

### A SOLDIERS' BAZAAR.

The Bazaar in aid of the convalescent Guardsmen at Golder's Hill, Hampstead, on Saturday, was very successful. Baskets, rugs, and toys, found a ready sale, large useful articles, such as linen-baskets, egg-baskets, hearthrugs, etc., being in special demand. A good many fretwork paper-knives and other small things were made by a soldier who has lost his right arm; and there are some particularly sad cases now in the home, to whom the money realised by the bazaar —about £50—will be a real help. Miss Arnold, the matron, is determined to sell the few things remaining over; only the cost of materials is deducted, the balance going to the convalescents. The matron was assisted at the bazaar by the resident nurse and many ladies living in the neighbourhood.

### THE MIDWIVES ACT.

At the opening meeting of the seventy-fifth series of the General Medical Council on Tuesday, Sir William Turner, the president, referred at length to the Midwives Act, which, as he pointed out, confers statutory recognition in England and Wales to an order of obstetric practitioners who have not hitherto possessed a legal position and title. The Act, he continued, with certain specified exceptions, will come into operation in April next. Summarising its features, Sir William said that it "made provision for the creation of a class of certificated midwives, the certificates to be conferred after a course of training and the passing an examination, and enacted that those who had been certified under this Act should be entitled to have their names entered on an official roll of midwives. It also made it penal after April 1905 for any woman to take or use the title of midwife, or to state that she was recognised by law as a midwife, unless she was duly certified under the Act; while from and after April 1910 it would be penal for any woman habitually and for gain to attend women in childbirth otherwise than under the direction of a qualified medical practitioner, unless she was duly certified under the Act." On Wednesday the official announcement was made that the Central Midwives' Board has been fully constituted. The two members added by the Lord President of the Council are Dr. W. J. Sinclair, of Manchester, and Miss Jane Wilson, President of the Midwives' Institute.

### THE SUPERINTENDENT NURSE AT DUDLEY UNION INFIRMARY.

The unsatisfactory state of affairs at Dudley Workhouse Infirmary is shown by the fact that the Local Government Board have had to express their regret that the master of the workhouse did not "realise the expediency" of consulting the superintendent nurse as to the condition of a man who died soon after his admission. But how can it be expected that the master will treat the superintendent nurse as he ought to treat her so long as the Dudley Guardians do everything they can to render her position difficult? We observe, however, that they do not seem likely to succeed in their attempt to force her resignation.

### ROYAL BRITISH NURSES' ASSOCIATION.

H.R.H. Princess Christian will open and preside at a Supplementary Sale in aid of the Royal British Nurses' Settlement to be held at 10 Orchard Street, W., on Tuesday next, from 3 to 8 p.m. Members of the association will be admitted free on signing their names. The sale will be open also, admission free, on the following day from 11 a.m. to 6 p.m.

### A GRUDGING RECOGNITION.

Crediton Guardians have had the usefulness of the District Nursing Association forced upon them. They have never been able to see the force of the argument that they should make an annual contribution to the funds, but recently when the workhouse nurse was incapacitated, the district nurse acted the part of the good Samaritan to the inmates by paying about 30 visits to the Workhouse Infirmary. The question of paying something in recognition of the services rendered has just received consideration by the Board on a proposition that three guineas should be voted. Those who advocated this payment were careful to point out that it did not represent the monetary value of the nurse's services. There was, unfortunately, a good deal of quibbling about it, and various suggestions were advanced as to how the money should be paid—whether as a donation, in consideration of kindness shown, or as remuneration for services rendered. But the Rev. R. Knight very rightly observed that if the Association presented a bill under the latter heading, on the ordinary scale of charges three guineas would not meet it. Eventually, as a sort of compromise, it was agreed to give the sum "in partial payment" of services. There should of course have been no hesitation in voting the sum. If the Guardians had been compelled to call in the services of a trained nurse from a hospital or nursing home for temporary duty on a similar occasion, they would have had a better idea of the cost of such assistance and a deeper sense of their obligation to the Nursing Association for coming to their aid in an emergency.

### AN UNREASONABLE DEMAND.

A demand for an inquiry by the Local Government Board at Blackburn has, we think, been properly negatived. The case was that of a probationer on trial for two months who, according to one of the Blackburn Guardians, protested that she had not been given the same opportunities for learning as the other probationers. But several Guardians demurred to this view, and the officials said that when the girl was asked by them if she had any grounds for complaint to make as to not having had a fair chance as regarded lessons and books, she replied that she had none. In any case, the work of the Local Government Board would never be finished if they had to investigate the reasons why the matrons of the Poor Law infirmaries under their control find would-be probationers unsuitable for their position.

### OUR CHRISTMAS DISTRIBUTION.

We have to acknowledge with many thanks the receipt of parcels of useful clothing as follows:—Nurse Cave Browne-Cave, Ennox Hill, Frome; J. G., Edinburgh; A Constant Reader; Policy 1,081; Miss Evelyn M. Smith, 3 Mortimer Villas, North Finchley; and Miss Maud Ruel, Hope Villa, Ryde, who also sends a shilling with the hope that every reader will do the same. The great need of these acceptable Christmas gifts for patients in hospitals and infirmaries does not, we are sure, require to be further impressed upon our readers. But we may remind them that Monday, December 15, is the latest day for sending in parcels, and that the name and address of the sender should be enclosed in each. All contributions should be directed to the Editor of The Hospital, 28 & 29 Southampton Street, Strand, London, W.C., marked "Clothing Distribution."

### SHORT ITEMS.

The *Scot*, which arrived at Southampton on Saturday, had on board Nursing Sister G. A. E. Napier, of the Army Nursing Service Reserve.—At their last meeting the Newton Abbot Board of Guardians considered tenders for the erection of the new home for the nurses of the institution. This work is to be completed by Midsummer next, at a cost of £1,088.

### Lectures to Nurses on Clinical Chemistry.

By Carstairs Douglas, M.D., B.Sc., F.R.S.Edin., Professor of Medical Jurisprudence and Hygiene, Anderson's College Medical School, and Dispensary Physician, Samaritan Hospital, Glasgow.

### LECTURE I.

It is a necessary part of a nurse's education that she should know something of clinical chemistry, that is to say, of certain chemical processes employed in practical medicine in order to help one to a true estimate of the nature, course and probable or possible termination of an illness. In all the hospitals in which nurses undergo training they receive instruction in this subject in their lectures, but I have often found that very intelligent nurses, though quite capable of carrying out tests practically, held very vague ideas as to the principles on which these reactions are based. Now it has always appeared to me that the knowledge of how a test is to be performed is scarcely more important than the reasons why such and such a procedure should be adopted; if a nurse or anyone else is really to do her work with intelligence, her knowledge of this matter must be a happy combination of the practical and the theoretical, and it is this which I shall attempt to give you now.

### The Chemistry of the Urine.

The most extensive use of chemistry from the clinical standpoint is in the examination of the urine, and rightly so, for this is a fluid excretion which can be very readily obtained, and is, moreover, one which is profoundly modified not only by disease of the kidneys themselves, but also by illness affecting other parts of the body. For instance, in such affections of the kidneys as acute or chronic inflammation (Bright's disease), cancer, and tubercle, we find important alterations in the urine, and the same holds good of such diseases as diabetes, pneumonia, and pernicious anæmia, in which the kidneys themselves are not specially involved.

*Collection of Samples.*—The urine varies a good deal in composition at different times of the day, being concentrated and very acid in the early morning, and more copious and much less acid after a meal. Now, if one wishes to be very particular, a sample chosen should be one from the mixed urine of the 24 hours. But even in hospital all the urine of all the patients is not usually collected, and I would recommend, if you are simply going to keep one sample for the physician, that it should be one passed in the forenoon or about two hours after breakfast. Such samples should be kept in "specimen"—or "urine-glasses"—cylindrical glass vessels on a solid flat base, and about 6 inches high. They should have a spout for pouring from, and the lower fourth of the interior should taper down to a point for the better collection of sediment. It usually takes three hours at least for a deposit to settle down fully.

*The Reaction of Urine.*—So far all has been plain sailing, but we now come to a point involving some chemical questions. I presume every nurse knows that healthy urine is acid, and she ascertains this by dipping into it a strip of paper coloured blue by a vegetable dye named litmus; if it is acid she notices that the blue tint is replaced by a red one. If the blue paper does not change, she says to herself that the specimen is either neutral or alkaline, and to find out if it is the latter or not, she uses a strip of red litmus paper, which becomes blue in an alkaline fluid. If the nurse should happen to ask what it is that makes urine acid, she is told that it is the presence of acid salts in that liquid, but if asked in turn what is meant by an acid salt she cannot very well explain. This brings us therefore to the question, what is really meant by the words "acid," "acid salt," and similar expressions. Now it is not very easy to say in precise terms what an acid is, without going too much into technical chemical terms, but in general we designate by the word "acid" certain substances which have a sharp biting taste, which can change the colour of certain vegetable dyes (for example, make blue litmus red), and which, if dissolved, impart to the finger a slightly sticky feeling, like very hard water when the solution is dilute, while the strong and pure acids will actually burn the skin, producing a feeling of smarting and pain. Some acids (not many) are gaseous, *e.g.*, carbonic acid; a great many are liquids, such as sulphuric, acetic, and nitric, while a good many are solids, as boracic, tartaric, and so on.

We now proceed to consider the antithesis of an acid, and that is a body termed a "base." For all practical purposes, all the metals such as copper, iron, magnesium, sodium, and so on, unite with the gas oxygen (the most important constituent of ordinary air) to form bodies called "oxides" of the metals, and all bases are of this nature—they are oxides of a metal. Some of the bases have very marked characters, and these are the oxides of sodium, potassium, calcium, magnesium, etc., familiar to us all by the names soda, potash, lime, and magnesia. These well-marked bases are grouped together under the name of "alkalies," and they have properties precisely the opposite of the acids, for they have a harsh caustic taste, they act on litmus paper so as to turn the red form blue, and their solutions have a very distinct soapy feel. Bodies in which these special bases occur are said to be "alkaline."

We now understand in a general way what an acid is, and also what a base is, and further what are the special characteristics of those bases named the alkalies. And these bodies, acids and bases, unite to form a third order of substances called "salts," which present the marks of neither acids nor bases; they do not (if they are what is termed "normal") affect either blue or red litmus paper, and their taste is "salt" rather than acid or alkaline. Salts always occur in the solid form, and are usually crystalline. To every one of you there will occur at once the names of many salts, such as sodium chloride or common salt, formed from soda and hydrochloric acid; saltpetre or nitrate of potassium, formed from potash and nitric acid; citrate of iron, formed from oxide of iron and citric acid, and so on. In fact, if acids be represented as bachelors and alkalies as spinsters, salts would stand for married couples. It has been known for a century at least that it always required a certain definite amount of acid to unite with a definite amount of base to form a salt, and these definite amounts were termed "equivalents." One equivalent of sulphuric acid unites with one equivalent of soda, for example, to form sulphate of soda (familiar enough under the name of Glauber's salts), and such salts are called "normal." But it sometimes happens that two equivalents of acid will unite with one equivalent of a base, and the resulting body is called an "acid salt." It partakes partly of the character of an acid and can turn blue litmus red, and it is owing to the presence of this kind of salt that urine is acid. Lastly, it may happen that one equivalent of acid unites with two equivalents of base (like a marriage, so to speak, in a Mormon community), and then we have a "basic salt," partaking to some extent of the characters of a base. These explanations will I trust clear up certain points in chemical nomenclature with regard to which you may have been in doubt.

### The Nurses of Kensington Infirmary.

INTERVIEW WITH THE MATRON. BY OUR COMMISSIONER.

There is one feature of special interest in connection with St. Mary Abbot's Infirmary at Kensington. It has attached to it a maternity training school. Moreover, credit belongs to the guardians of the parish for taking this step at a period when the importance of midwifery training was far less fully recognised. There have been midwifery probationers at Kensington Infirmary since 1889. When I paid a visit to the institution in Marloes Road the other day for the purpose of having a chat with Miss Malim, the matron, she was good enough to show me not only some of the ordinary wards and the nurses' quarters, but also to enable me to see for myself the accommodation provided in the special department which is in charge of a highly-qualified midwife. The labour wards are roomy and comfortable, entirely separate from the workhouse, and the probationers have their own rooms as well as their own wardmaids, quite apart from the nurses of the infirmary.

### The Midwifery Pupils.

"In fact," as the matron observed, "the sick in these wards are entirely under the medical superintendent, Dr. Potter, who has always taken the greatest interest in the work, while I am responsible in regard to administration."

"You have, I see, only two wards with ten beds in each. Are they sufficient?"

"Yes, as regards the patients; but we could take more pupils if we had accommodation for them."

"Then I conclude that you are not able to take more than a limited number of midwifery probationers?"

### Rush of Applicants.

"We can only take eight pupils during the year. Applications have consequently to be refused in large number. Since the Midwives Act was passed they have greatly increased, and at the present moment we are full up for two years."

"What are the conditions on which the probationers are received?"

"They have to pay a fee of £10, which includes board, lodging, laundry, and medical attendance if necessary. The period of training is three months, or until the pupils have seen the number of cases required by the London Obstetrical Society. In order to insure the perfect administration of the ward, all the nursing and minor ward cleaning is performed by the midwife and her pupils. I visit the wards every morning. The midwife is a fully-trained nurse, and receives a salary commencing at £40 a year, and rising to £45."

### An Essential Condition.

"Is it essential that the pupils should also be fully-trained nurses?"

"Yes; at one time it was not a rule that they should have received the three years' training, but when the Midwives Bill became law, it was considered requisite to make it so; and now it is useless for anyone who has not been trained for three years in one of the general hospitals or infirmaries to make application for midwifery training here. We get a very good class of candidates from all the leading London and provincial hospitals, and, in fact, are able, owing to the number of applications, to choose the best."

### The Age Limit.

"Is there a limit as to age?"

"It has been found necessary to impose one. We do not receive a pupil under 25 nor over 35. The average age of our pupils is from 28 to 32."

"Do the ordinary rules and regulations for the nursing staff apply to the midwifery probationer?"

"Generally speaking, they do, though there is a special set of rules to which it is their duty to conform. As to the training, lectures are given them once a week by the medical superintendent and by the midwife. The latter prepares the pupils and the former sets them their papers."

"Do they all pass the examination of the London Obstetrical Society?"

"Mostly. I certainly strongly advise nurses to go in for the L.O.S. certificate. Many fail, and the examination is getting more strict every year; but maternity training has now become almost indispensable, and for those who desire to undertake private nursing abroad or in the colonies it is of the first importance."

### Growth of the Nursing Staff.

"As to nursing generally, you have had a training school for some years?"

"Since 1891. The greater part of the infirmary and the workhouse was built in 1872, but the male ward of the latter was added in 1890. Miss Frances Hughes formed the training school for three years, but I was one of the first sisters, and subsequently as assistant matron assisted in its development. I have been matron between five and six years."

"I conclude that the staff has been considerably augmented?"

"In the days before there was a training school there were 26 assistant nurses. Now there are 16 sisters and 49 probationers."

### The Superannuation Act.

"At what age do you receive probationers, and what salaries do the Guardians offer?"

"Applicants must be between 21 and 30, and single. The salary is £12 the first year, £15 the second, and £18 the third. If a probationer desires to avail herself of the provisions of the Poor Law Officers' Superannuation Act of 1896, a deduction is made at the rate of £2 per cent. on the salary and the value of the board, lodging, etc., estimated at £20 per annum. In such a case the deductions are 15s. 2d., 16s. 4d., and 17s. 7d. in each of three years respectively. In return for this contribution she will be entitled to a superannuation allowance under the conditions and in accordance with the scale prescribed in the Act."

"Must a probationer give any notice if she does not wish to avail herself of the provisions of the Act?"

"Yes, at the end of two months from the date of her appointment she must signify on a form which is supplied, but in that case she does not of course obtain any of the benefits of the Act. Moreover, she cannot at any future time avail herself of it, either under the Kensington Guardians or any other authority to whom the Act applies, because she will have 'contracted out.'"

### Uniform and Hours of Duty.

"Probationers" continued the matron "come on trial for two months, and if at the end of that time I find they are suitable, they are required to sign an agreement to serve for three years, dating from their entry on duty. They are then supplied with uniform."

"For indoor wear?"

"Only indoor uniform is provided gratuitously, and therefore it is not compulsory to wear uniform out of doors. But we have a regulation uniform, consisting of dark blue bonnet

THE NURSES OF KENSINGTON INFIRMARY—*Continued.*

and cloak, which we expect nurses who profess to be in uniform out of doors to wear; and, as a matter of fact, the majority wear it, because it is so easy to slip on and off."

"I should like to know the hours of duty?"

"Probationers on day duty rise at 5.45 A.M.; breakfast at 6.15; go on duty at 6 45; dine at 1.30; have tea at 5; go off duty and have supper at 8. Those on night duty leave the bedroom at 4.30 P.M.; have an evening meal at 7.15 P.M.; morning meal at 8.15 A.M; and go to bed at 9 A.M. Half an hour is allowed for each of the meals."

"When do the probationers on night duty go out?"

"Between 4.30 and 7 P.M. They prefer this time both in winter and in summer. The day sisters enter the wards an hour later than the probationers, but their hours are similar. The whole of the staff have a half-day off duty each week, four hours on alternate Sundays, and from 2 P.M. to 4 P.M. twice a week. Nurses on night duty have a whole day once a month as well as two-and-a-half hours daily. Each probationer does eight months' day work and four months' night work, but no one is put on at night before she has had six months' experience. The sisters have three and the probationers two weeks' holiday."

LECTURES AND EXAMINATIONS.

"What about lectures?"

"There are three courses, the first on the general details of nursing, by myself; the second on elementary anatomy and physiology, by the assistant matron, who also delivers a course of instruction in practical bandaging; and the third by the medical superintendent on nursing and hygiene. At the end of their third year the nurses go up for their final examination by an independent examiner — Dr. Seymour Taylor of Wigmore Street. The 'finals' are in May and November. The advantage of an independent examiner is that as he does not know any of the nurses he cannot be otherwise than quite impartial."

"By whom is the certificate at the end of the training signed?"

"By the Examiner, the Lecturers, and the Chairman of the Board of Guardians. It is simply to the effect that the nurse has completed three years' training in the Infirmary and that she has successfully passed the medical and surgical examination rendering her competent to practise as a certified nurse."

SALARIES AND VACANCIES.

"You do not append any remarks."

"No, but before engaging a Sister I always write to the matron of the institution where she was trained, and matrons engaging any of the nurses from this infirmary write to me. After the full term of training here probationers, who elect to stay, are promoted as vacancies occur, to the post of sister, with a salary of £30, rising £2 10s. annually to £35, and £3 10s. a year in lieu of beer. I keep a record in which the names of probationers are entered and their conduct and progress recorded. This can always be referred to in case of future inquiry."

"Do you have a sufficient number of applications for probationership?"

"As a rule I have enough names on the books. But once a year I require to advertise. I have just been advertising in THE HOSPITAL and I have received hundreds of applications for three or four vacancies. Formerly, we did not accept probationers who had been trained anywhere else for a year, but if a girl seems suitable and is willing to begin from the beginning and drop into our system of training, I do not object to her."

"Is the class of applicants good?"

"I am able to choose a good class. We are proud of the fact that several Kensington nurses did well in South Africa. Sister Hogarth, who was recently awarded the decoration of the Royal Red Cross, was trained here."

THE NURSES' QUARTERS AND RECREATION.

"You have separate blocks for the accommodation of the nurses?"

"Yes; the night nurses and the probationers have quarters of their own, and the sisters' rooms are in the administration block close to me. The night superintendent is in charge of the night nurses, and the assistant matron of the probationers. They all have general sitting-rooms, and the sisters have separate bedrooms."

"But not the probationers?"

"Unfortunately there is not sufficient space to give the probationers a separate room. But their separate cubicles are made as comfortable as possible. The nurses are allowed to see their friends in the recreation rooms, between 2 and 8 P.M."

"Have you anything to say on the subject of recreation?"

"I like the nurses to go to matinées and concerts. The perhaps inevitable tendency is for them to regard the infirmary as the only place of interest, and I think it is good for them and good for the patients, that they should go outside occasionally and forget all about the institution.

### A Case of Simple Fracture of the Leg.

### EXAMINATION QUESTIONS FOR NURSES.

THE question was as follows:—"Imagine yourself called to assist a doctor in a lonely cottage miles from town. The case is one of simple fracture of the leg. Not having been told it is a case of fracture, the doctor has brought no splints. What would you do to help him?"

FIRST PRIZE.

If the case was one of fractured femur, I should look about for a piece of wood about 4 feet long, and from 3 inches to 5 in breadth, out of which a splint might be made. A lath from a four-post bedstead for instance. Failing this an ordinary long broom might be pressed into service; the brush being wrapped in some old linen might be useful to keep the limb steady if sandbags were placed over it in its position as foot piece. Bags filled with small stones, flat irons, or weights tied together might be used if sand were not at hand.

As it would be difficult to get wool, peat, or tow in the country, I should make some small firm pads filled with bran or even hay, and also have some pads of old linen at hand.

Bandages might be made from old linen (a sheet for preference); and a round towel or even a hand towel might be useful to fasten the splint to the trunk of the patient.

If the fracture were of the lower leg, three pieces of wood from 3 to 5 inches in breadth and 24 inches in length might be useful, and failing these folded newspapers would make a temporary splint.

If the patient were on a feather bed, I should remove it if possible, and if not, should have as few feathers as possible under the injured limb, making the bed firm and level with a folded blanket or two.

If on a spring mattress I should place boards over the springs under the mattress if possible, and failing these I should use anything that would keep the bed straight and the leg steady.

Two small wooden boxes of strong make might be useful to raise the foot of the bed, failing these bricks or even books might be used.—"Cassy."

Second Prize.

Search the back premises, wash-house or outhouse for old wooden boxes frequently obtained when empty from the grocer. If any are found they can easily be chopped into splints the required length and width with the wood chopper which one may certainly count on. Failing wooden boxes perhaps cardboard or millboard boxes may be forthcoming. Cut these up and several layers used for each splint would answer well. Laths out of the window blinds might be used to strengthen or altogether take the place of the above, but would require a considerable amount of padding to be of real use alone owing to their narrow width.

For padding use first of all layer upon layer of paper—newspapers, illustrated papers, magazines. Look for these in the front parlour. The poor do not part with such things when read. Old book-covers might be converted into splints. Complete the padding with pieces of old blanket, cloth, flannel or shawl, a chair-cushion and window-blind laths strapped round the limb with webbing braces, if bandages are not to be had, would keep the limb at rest till something a little less primitive could be obtained. Whatever make-shifts were used they would probably be replaced with something more suitable on the doctor's next visit. If near sand-pits, or the seashore, sandbags could be made by emptying a pillow of its stuffing, running two rows of close firm stitching about six inches from each end across the pillow and filling the divisions at the ends with thoroughly dried sand. The empty centre-piece would lie under the limb, the sandbags at either side kept in place by a towel enclosing both sand-bags and the leg fastened with safety-pins or sewing.

By means of a packing needle, a piece of twine can be drawn through the bedclothes covering the patient, and tied to the foot of the bedstead thus relieving pressure by slightly elevating the clothes.—Damaris.

A Good Standard is Reached.

The question this month seems to have been a popular one, and the generality of the answers are good, decidedly above the average. "Cassy," who gains the first prize, is among the few who recognises that a fractured leg may mean any part of the limb. The question was framed with a view to testing the nurses' aptitude for comprehending a plain question put in plain language. Clearly understand that a fractured femur must always be a broken leg, though a broken leg is not always a fractured femur. It seems nowadays that increased knowledge of technical terms has sometimes a tendency to obscure common sense.

"Cassy" recognises the insuperable difficulty of the feather bed, too, and does what is best as a makeshift until more drastic measures can be employed.

"Damaris" has some good ideas, and I like to note that she is a person of observation and has taken stock of the dusty piles of antique papers hoarded in the horrid best parlours. "Look for these in the front parlour," she says. Quite right, the accumulation of years abide there in company with wax flowers, the Family Bible, mourning cards, and wool mats.

Honourable Mention.

Four nurses have gained this:—"Betty," "Corubia" (is this name correct?), "Caustic," and "Niggs."

I think "Betty" could hardly carry sufficient padding in her bag for emergencies, the bag would need to be indeed capacious.

"Caustic" and "Betty" have been successful before.

A Striking Piece of Carelessness.

A question was inserted in October for the benefit of our nurses in the colonies and abroad generally; six months were allowed for the answers to be sent in.

No less than six nurses at home have answered this. It is very careless, and gives much unnecessary trouble.

The Examiner.

Question for December.

What steps would you take to isolate a case of infectious disease in a private house to prevent the spread of infection?

N.B.—Consider cases of scarlet fever, small-pox, diphtheria, and enteric.

### Everybody's Opinion.

**[Correspondence on all subjects is invited, but we cannot in any way be responsible for the opinions expressed by our correspondents. No communication can be entertained if the name and address of the correspondent are not given as a guarantee of good faith, but not necessarily for publication. All correspondents should write on one side of the paper only.]**

WHOLESALE DISMISSAL OF NURSES.

"One of the Dismissed Nurses" writes; Regarding the wholesale dismissal of nurses from the Cheltenham Hospital, it may be of interest to your readers to see the actual wording of the petition stigmatised by Mr. Carrington as both "offensive" and "dictatorial" affixed below. The nurses are only too conscious that, as regards wording, the petition leaves much to be desired; but it was written under great excitement and a smarting sense of injustice, and no act of insubordination was intended. Mr. Carrington's letter gives the idea that none of the dismissed nurses had been in the hospital for more than three years, whereas three had been in the hospital over five years. Moreover, of the thirteen, four hold a three years' certificate, obtained at the Cheltenham General Hospital, one a three years' certificate, and another a four years' certificate obtained elsewhere. With respect to Mr. Carrington's statement that four of the nurses were subsequently retained, as it was considered that their action had been "forced upon them by their seniors," and that the signature of another had been affixed without her consent, I may say that one of the four referred to is one of the seniors on the staff, and the nurses who have been dismissed declare in self-defence that they certainly did not force any-one to join them in signing the petition. "The signature affixed without the nurse's consent" was written in consequence of a *bona fide* mistake, which might be explained in detail, but was not the fault of the nurses. The nurses, Mr. Carrington says, have "received the certificates to which they were entitled," but not the usual testimonials, and they were given to understand at first that the certificates would not be signed. The nurses did send an apology, which was not accepted except in the case of the four nurses retained. In conclusion, the nurses fail to see that the fact that they have "nearly all obtained posts elsewhere" has any bearing upon the action of the committee in so summarily dismissing them without giving them a hearing.

Copy of the Petition sent to the Committee.

The General Hospital, Cheltenham, October 1902.

Dear Sirs,—We, the undersigned private nurses, ask the committee for an explanation of the appointment of the sister of Ward 8. We feel both puzzled and insulted that a nurse should be chosen out of the hospital staff with her three years' training unfinished, and with a salary of £16 to be suddenly raised to that of £30, while we have served the hospital many years, all more than three years. In the past the sisters have all been chosen from the private staff. Surely one capable and suitable person can be found out of 19 nurses? We also feel that after having served the hospital faithfully for so many years (as our reports will show), in expectation of promotion when opportunity should offer itself, that we must request either a salary equal to that of the sisters, viz., £30 and uniform, with also the percentage on our earnings as formerly, or simply justice in the appointment of one of our number to the vacant post in ward 8. Otherwise we are all prepared to send in our resignations. We greatly regret having to take so decided a step, but the circumstances enforce it.—We are, sirs, faithfully yours—

"Champion" writes: Referring to the letter by the Honorary Secretary of the General Hospital, Cheltenham, in your issue of November 22nd, I beg to say as one who has a personal knowledge of the case, that the explanation given is unsatisfactory and misleading. Most certainly 14 nurses received notice of dismissal on October 14th. A day later, a message was sent to them intimating that if an apology was not forthcoming, they must be prepared to forfeit their certificates. At this intimidation, ten nurses sent in a joint apology. Out of this number four were accepted and six rejected. One of the four, whose apology was accepted, decided not to remain, and left on the 14th inst. with her fellow nurses, thus making ten who left the hospital on that date. Three nurses were left on the staff, and one away at a fever case who had telegraphed her sympathy with the petition which was being prepared to be sent in to the committee, and requested that her name might be attached. Strange to say, the nurses who are retained were the most zealous in wishing that the petition should be forwarded; I am not aware that it has been stated that fourteen nurses have left the hospital, but that was certainly the number who received notice of dismissal. I believe there was a half-hearted offer of the post as sister given to one of the senior nurses, who declined it. Several of the nurses who signed the petition, after having passed through the necessary three years' training, were only receiving £20 yearly salary and a small percentage on their earnings, with uniform. I note that the honorary secretary of the General Hospital refers to the newly-elected sister as "assistant nurse." Whatever that may infer —I speak with all due respect to the lady in question—she came to the hospital as a probationer, less than three years ago, and, I take it, she remains a probationer until that time has expired. It is true that the nurses were given their certificates of training on leaving the hospital, their legal due, but though good reports of their capabilities and conduct were sent to the hospital by friends of the patients they had nursed, no testimonials were given them. I believe that most of the nurses, since leaving, have obtained more lucrative appointments than they formerly held, but small thanks are due to the ruling powers at the Cheltenham Hospital. One of the nurses lost a most desirable appointment by referring to Cheltenham for a reference.

### NURSING HOMES IN MARYLEBONE.

"Yorkshirewoman" writes: Will you grant me a little of your valuable space in which to refer to the note on the "Multiplicity of Nursing Homes in Marylebone." Several of my fellow nurses feel with me that such a palpable betrayal of utter selfishness and extreme indifference to suffering humanity, as the protest alluded to should not go unchallenged. Really, one might imagine this "protest" was directed against some body of people who wilfully deposited themselves near and around a "pleasure-seeking world" with intent to make themselves a nuisance and annoyance to those who know no higher aim in life than enjoyment of its fleeting pleasures, instead of against those who, at considerable self-sacrifice, have joined in the great crusade for the alleviation of human suffering. Sacrifice both on the part of those who provide the "homes" for patients, and of the nurses who give up liberty and home and friends, toiling through their "training" to secure a well-earned certificate of efficiency, and in many cases a ruined constitution. The mention of "ex-barmaids" and "domestics" is incongruous in such a connection. Where is there one of either class who would unceasingly perform the duties that fall to a nurse, under the same conditions? Obviously, the "very strong protest" comes from those who have never known serious illness, or the dire extremity of despair that calls for prompt and skilful operation, and, it would seem, never expect to, or they would more charitably regard the provisions made for these contingencies. One might almost as reasonably "protest" against the poor patients for developing the various "ills that flesh is heir to," and I am sure both patients and nurses, as well as the fastidious "residents" in Marylebone, would be eternally grateful for any suggestion by which to avert the "inhalation of noxious odours emanating therefrom." But to all sensible-minded people this will be regarded as a "necessary evil"—a natural sequence—to the existence of disease. Remove the cause—the effect will also be removed. As to the "hearing of groans from patients under operation"; I should like to hear of any nurse who has been present at any operation where the patient "groaned" during that critical performance! Speaking from my own experience there has always been a death-like silence, only broken by the click of the surgeon's instruments, or an occasional whispered instruction; and often for hours after this a patient's life may depend upon sleep, to try and secure which is inflicted upon the public the laying down of "malodorous and putrefying straw" which, alas! entails the spoiling of "box-calf" or "glacé," or it may be the more important silk frills! The disturbances to patients in these "homes" from neighbouring private residences count for nothing, I suppose"; for instance, where on one side of a "home" where one lay dying and others seriously ill, a constant strumming of a high and harsh-toned piano was kept up all day, whilst all night on the other side five young pups indulged in a concert of yelping and barking and whining immediately under our windows. Apropos of the "groans" I should like to relate that about two months ago I entered upon the staff of a nursing home in Marylebone. During the whole of that time I was distressed beyond measure by hearing the most piteous cries and groans issuing from an adjoining private house during the greater part of each night. One night the sounds were so harrowing and unbearable that at last I went down to the street door shortly after 3 A.M., and, hailing a passing P.C. and assuring him that my questioning was not from idle curiosity but from real concern, asked if he could explain the sounds. He informed me there was a chronic sufferer from rheumatic gout in that house, absolutely helpless, and a perfect martyr. Now, if this had gone on month after month in a "home" (for every previous night nurse had heard the same and declared the house was haunted), residents would have cried shame! and, not unjustly, have uttered "protests." But in any "home" something would be done to secure sleep for such a sufferer. "Pups" and "pianos" etc. may "enhance the pleasures of life" for their owners, but they are not conducive to success in the work which is actuated by a desire to fulfil the "Golden Rule."

### Appointments.

[No charge is made for announcements under this head, and we are always glad to receive, and publish, appointments. But it is essential that in all cases the school of training should be given.]

Aylsham Workhouse.—Miss White has been appointed superintendent nurse. She was trained at the Leicester Infirmary, and has since been first assistant nurse at the North-western Fever Hospital, London, charge nurse at Romford Infirmary, staff nurse at Camberwell Infirmary, and head nurse at St. George's Infirmary, Fulham Road, London.

City Hospital, Lodge Moor, Sheffield.—Miss F. Crichton has been appointed assistant matron, and Miss E. L. Chippindall night superintendent. Miss Crichton was trained at St. Bartholomew's Hospital, London, has been home sister and night superintendent at the Birmingham General Hospital, and has served in the Royal Naval Nursing Service. Miss Chippendall was trained at Kensington Infirmary, and has since held appointments at the South-western Fever Hospital, London, and the Brook Fever Hospital, Shooters Hill. She has also been superintendent nurse at Dorking Infirmary.

Consumption Sanatorium, Heswall, Cheshire.—Miss Foxcroft has been appointed sister. She was trained at Mill Road Infirmary, Liverpool, and has since been staff nurse at the Hospital for Diseases of the Chest, Brompton, London.

Eastville Workhouse Hospital.—Miss Helena Jane H. Dobbins and Miss Florence A. Branston Smith have been appointed charge nurses. Miss Dobbing was trained at Guy's Hospital, London, and Boston Hospital, Lincolnshire. She has since been nurse at Hartlepool, West Derby, and other Poor Law Infirmaries. Miss Branston-Smith was trained at the Chelsea Infirmary, and was subsequently staff nurse.

Hendon Isolation Hospital, Great Stanmore.—Miss Allison Streeter has been appointed nurse-matron. She was trained at Brompton and St. Thomas's Hospitals, London, and has since been ward sister at Wimbledon Isolation Hospital.

Kensington Infirmary.—Miss Beatrice B. W. Lewsey has been appointed sister. She was trained at Poplar and Stepney Infirmary, and has since held an appointment at the Royal United Hospital, Bath.

Kent Nursing Institution, West Malling.—Miss Kelly has been appointed lady superintendent. She was trained at the West Kent General Hospital, Maidstone; and the London Hospital, Whitechapel.

King's Norton Union Workhouse Infirmary.—Miss Fanny Helen Barlow has been appointed sister, and Miss Margaret Birkett maternity sister. Miss Barlow was trained at Birmingham Workhouse Infirmary, and has since been sister at Portsmouth Union Infirmary. Miss Birkett was trained at Middlesbrough Union Infirmary, and has since been charge nurse and temporary charge night nurse in the same institution.

Nottingham City Isolation Hospital.—Mrs. Eliza Aline Bouverie has been appointed sister. She was trained at the Middlesex Hospital, and afterwards at the South Tottenham Fever Hospital, where she became sister. She subsequently was charge nurse in the enteric wards of the Base Hospitals at Capetown, South Africa.

Rochford Infirmary.—Miss M. A. Foster has been appointed superintendent nurse. She was trained at St. George's Infirmary, Fulham Road, London, and has since been ward nurse at Camberwell Infirmary, and sister at St. Pancras Infirmary. She holds the L.O.S. certificate.

Rochford Workhouse Infirmary. — Miss Maud Alexandra Foster has been appointed superintendent nurse. She was trained at St. George's-in-the-East Infirmary, London, and has since been sister-in-charge at St. Pancras Infirmary.

Royal Masonic Institute for Boys, Bushey, Herts. —Miss Hilda Mary Good has been appointed matron. She was trained at St. Thomas's Hospital, London, after which she worked for the Queen's Jubilee Institute for a year and a half. She has since been at University College Hospital for the last three years, the greater part of the time as housekeeping-sister.

Swansea General and Eye Hospital.—Miss Prudence Crispin has been appointed matron. She was trained at St. Marylebone Infirmary, where, as sister, she afterwards took charge of a floor of 62 beds. She has also been assistant matron at Paddington Infirmary.

Victoria Infirmary, Northwich.—Miss Mabel Thornton has been appointed staff nurse. She was trained at Leeds General Infirmary.

### To Nurses.

We invite contributions from any of our readers, and shall be glad to pay for "Notes on News from the Nursing World," or for articles describing nursing experiences, or dealing with any nursing question from an original point of view. The minimum payment for contributions is 5s., but we welcome interesting contributions of a column, or a page, in length. It may be added that notices of appointments, entertainments, presentations, and deaths are not paid for, but that we are always glad to receive them. All rejected manuscripts are returned in due course, and all payments for manuscripts used are made as early as possible after the beginning of each quarter.

### For Reading to the Sick.

### ADVENT SUNDAY.

Thus to hail Thine Advent hither,
Grant, O Lord, to me
Large delight and griefs together
May united be:
Here though bitterness hath found Thee
For our guilt undone,
God's high Pæan fails around thee
For a conquest won.

Thus alternately to borrow
Health from pain and loss,
Joy's companionship with sorrow
Yield me from Thy Cross;
Tears for Thy deep Tribulation
And sin's wine-press trod,
Praise for uttermost Salvation
And the Hymns of God.

*P. S. Worsley.*

O Lord Jesus Christ, Who has promised to come at a time when we look not for Thee, and at an hour when we are not aware, mercifully grant that we, following Thy precepts and example in this life, may at Thy coming be found among the number of Thy faithful ones, evermore to dwell with Thee.—*Anon.*

O most glad hour, when it shall dawn towards the first day of the everlasting week, when there shall be a making ready in the heaven above and in the earth beneath; when legions of angels shall gather round the Sun of Righteousness, and all orders and hosts of heaven shall know that the time for "the manifestation of the sons of God" is come. What joy shall there be at that hour in the world unseen! and what a thrill, as of a penetrating light, shall run through the dust where the saints are sleeping! When was there ever such a day-spring since the time when "God said, Let there be light; and there was light"? He shall come and all His shining ones; ten thousand times ten thousand, whose countenances are "like lightning," and their "raiment white as snow," all the heavenly Court—angels, archangels, cherubim and seraphim —clad in unimaginable splendours; and the righteous shall arise from the grave, and the earth shall be lightened with their glory; they shall stretch forth their hands to meet Him, and bow themselves before the brightness of His coming. O blessed hour, after all the sorrows, and wrongs, and falsehoods, and darkness, and burdens of life, to see Him face to face; to be made sinless; to shine with an exceeding strength; to be as the light in which there "is no darkness at all"! Be this our hope, our chiefest toil, our almost only prayer.—*H. E. Manning.*

O Saviour, unto Whom are all things given,
Come with Thy voice of love and claim Thine own!
Good Shepherd—knowing all and known of all—
Oh come, and call Thy sheep from off the wild!
Monarch, in mercy and in power supreme,
Take for Thine own the kingdoms of the world!
God! Whose high thoughts and ways are over ours,
As yonder heaven sublime above the earth,
Come in Thine own good time; we wait for Thee!

*S. J. Stone, S. Augustine and Monica.*

### Echoes from the Outside World.

### Movements of Royalty.

THE King of Portugal terminated his visit to the King and Queen on Monday, after a week's stay, and proceeded to Didlington Hall, Norfolk, as the guest of Lord Amherst of Hackney. During the time King Carlos was at Windsor, he enjoyed splendid sport, and on Friday, after a long day's shooting, he witnessed a theatrical performance at the Castle in the evening. It is stated that he expressed a wish to see a Shakspearian drama, being himself an enthusiastic student of the great poet, and was much surprised to find that not a single one of his masterpieces was at that particular time being played in London. However, he appeared to vastly enjoy "Quality Street," and Mr. and Mrs. Seymour Hicks and Miss Marion Terry were afterwards presented to him, as well as to the King and Queen. Her Majesty interested herself more particularly in the 17 children, members of Mr. Steadman's choir, who appear in the play. A substantial meat tea had been arranged for them upon their arrival in the afternoon, and almost before the meal was ended the Queen and the Princesses came in and talked to them in a quite informal way for some little time. Later on, when the King and Queen learnt that the young folks were supposed to return to town with the rest of the company at 2 A.M., their Majesties expressed a wish that they should not do so, and they were accordingly accommodated in Windsor for the night, and shown round the famous town next morning before they went back to London. On Saturday the royal party were present at a musical ride at the Riding School, and on Sunday afternoon the King and the Prince of Wales showed King Carlos round St. George's, and the Albert Memorial Chapels.

### Mr. Chamberlain's Departure.

ON Tuesday morning Mr. Chamberlain, accompanied by Mrs. Chamberlain, left London for South Africa as the nation's ambassador. At his express desire the departure was of a semi-private character, but outside the entrance and in the courtyard of Victoria Station a large and enthusiastic crowd assembled. On the platform were a number of distinguished persons, including the Prime Minister and Miss Balfour, Lord and Lady Roberts, Lord Onslow, and several other members of the Government. An amusing incident occurred just as the train was moving. Sir Benjamin Stone, M.P., the famous amateur photographer, rushed on to the platform, without his camera, and shook hands with the Colonial Secretary amid much cheering. At Portsmouth Town Station an address was presented to Mr. Chamberlain, who briefly responded, and said that he would do all in his power to fulfil expectations in South Africa. The *Good Hope* left Portsmouth Dockyard later in the day, and is expected to arrive at Durban on December 21st.

### The New Paymaster-General.

THE King has approved of the appointment of Sir Savile Crossley, M.P., as Paymaster-General in place of the Duke of Marlborough. Sir Savile, who has also consented to act as one of the Government Whips, is one of the most popular members of the House of Commons. During the South African War he did excellent work with the Imperial Yeomanry and as a Lieut-Colonel of the Mounted Sharpshooters, his services being mentioned in dispatches. He represented the Lowestoft division of Suffolk in the House of Commons from 1885 to 1892, and since 1900 has been member for Halifax. The Paymaster-General does not receive a salary, but he is charged with the important duty of superintending the issue of all moneys voted by Parliament. Sir Savile Crossley is one of the honorary secretaries of King Edward's Hospital Fund for London.

### A Famous German.

HERR FRIEDRICH KRUPP, the great ironmaster, died at Essen on Saturday from an apoplectic seizure. He was the son of Alfred Krupp, who by means of an invention he discovered for casting steel in large masses, brought his firm—which began its existence with two workmen only—into the front rank of ironfounders. Although all sorts of steel articles were made at the foundry, the name is particularly associated with cannons, and Alfred Krupp made the gigantic siege guns which did such deadly work against the walls of Paris in the Franco-German war. All the offers of letters of nobility made by the German Emperor have always been steadily declined by members of the firm. More than a million people depended upon Herr Krupp and his foundry for their daily bread, and it is said that the head of the firm paid income-tax on a million sterling. His wife is his sole legatee, and when she dies his eldest daughter, now seventeen, will inherit the whole estate. Herr Krupp always took the greatest personal interest in the welfare of his employés. He pensioned widows and orphans, built a huge and handsome hotel at Kiel for the use of convalescents, and his workmen's hospitals are among the best in Europe. The Kaiser expressed his grief at the sad news, and attended his funeral on Wednesday.

### Cruelty to a Child

FOR two days last week Mr. Justice Bigham was occupied at the Central Criminal Court in trying a charge against Mrs. Annie Elizabeth Penruddocke, wife of Mr. Charles Penruddocke, J.P., of Compton Park, Compton Chamberlayne, near Salisbury, who was accused "of assaulting, ill-treating, and neglecting her child, Letitia Constance Penruddocke, in a manner likely to cause unnecessary suffering or injury to health." The child is the youngest but one in a family of five, and is just seven years of age. The servants employed at Compton Park and two governesses, deposed that they had heard screams from the child, whose body afterwards exhibited marks of ill-treatment. The mother had been seen to knock the little girl down, and she had been heard to say that "she wished she had fallen off the donkey and broken her neck." When a visitor admired the child's hair she at once had it all cut off. It was also stated that the child was once made to stand on the bough of a tree for four hours in inclement weather as a punishment; that she was ill-fed and clothed, and that she was made to eat cake with mustard and pepper spread on it. Moreover, on one occasion the mother had been seen to gather nettles with glove-protected hands, the child's neck and face afterwards showing signs of having been rubbed with nettles. The defendant and her husband denied all these statements, and said that the reason why the child was dieted, had few clothes on her bed, and was occasionally corrected with a twig, was because she had an infirmity, a bad habit of which she needed to be broken. Neither could in any way account for the severe weals and bruises which the doctor, upon examination, had found upon the child's body. The jury convicted the defendant of ill-treatment and occasioning actual bodily harm, and severely censured the husband for countenancing the ill-treatment. Sir Edward Clarke, defendant's counsel, asked that his client might not be sent to prison, and the judge, admitting that his sentence was not likely to meet with universal approval, merely imposed a fine of £50. This was at once paid, and defendant left the court. The sentence has occasioned many protests in the newspapers, and much indignation in Wiltshire, because of its leniency. The child is, for the future, to be placed under the care of her paternal grandmother.

### Notes and Queries.

**The Editor is always willing to answer in this column, without any fee, all reasonable questions, as soon as possible.**

**But the following rules must be carefully observed:—**

**1. Every communication must be accompanied by the name and address of the writer.**

**2. The question must always bear upon nursing, directly or indirectly.**

**If an answer is required by letter a fee of half-a-crown must be enclosed with the note containing the inquiry, and we cannot undertake to forward letters addressed to correspondents making inquiries. It is therefore requested that our readers will not enclose either a stamp or a stamped envelope.**

*Training.*

(63) Can you tell me where I could get five weeks' training in nursing during the Christmas holidays, north of England preferred. I am matron in a boys' school.—*Seeker.*

Your only chance of getting what you require is by advertisement.

*Holiday.*

(64) I have been given a month's notice because I claimed a week's holiday after 19 months' service. According to the rules I was entitled to eight days every year. When I asked for my holiday, the matron told me I could not have it, as I was a nurse "of no great shakes." I should like to know what other nurses think of my case. I may add that I had good testimonials from the medical officer and chaplain.—*A Mental Nurse.*

You may congratulate yourself that you have left the establishment.

*Needlework.*

(65) I am anxious to know if you could help me to obtain orders for fancy work, from a business-house, for a poor girl who is crippled for life, and who is very clever with her needle?—*District Nurse.*

There are often advertisements for needlewomen in the *Daily Telegraph*, but they may be from employers who live too far away from your *protégée* to be of use. If you both advertise and answer advertisements, you will probably succeed in time.

*Corns.*

(66) Can you tell me of any cure for corns? I have tried several, but without any effect.—*Nurse.*

For obstinate corns you had better consult a medical man.

*Child's Nurse.*

(67) Can you give me any information as to whether there is an institution for training ladies in the nursing of children's diseases, in order to fit them for the post of head nurse?—*F. D.*

You can obtain the training you require at any of the children's hospitals, assuming that you are eligible for admission as probationer.

*Maternity.*

(68) Will you kindly tell me what are the subjects given for the L.O.S.? Is it necessary to learn Latin? Where may I obtain full information?—*Nurse Lizzie.*

Apply to the Secretary of the London Obstetrical Society, 20 Hanover Square, W. No Latin is required.

*Meno* will be much obliged if the Editor will kindly tell her if the fact of seeing 21 maternity cases under a qualified practitioner and studying alone, would enable her to sit for the L.O.S. examination; and, if so, what book would he recommend her to study?

"Memo" would have to receive a course of theoretical instruction which would satisfy the Examining Board of the Society, from the practitioner before she would be eligible to sit.

I was engaged for a case from September 10th, but the baby arriving on August 22nd, I was engaged attending another case, and could not go until September 6th. I believe that the lady only wants me for the fortnight, and I should like to know if I should be justified in asking full fees for the whole of the month. I had refused a case on October 2nd as I expected to be engaged with this case until October 8th.—*C. S.*

This is one of the uncertainties that make the calling of a maternity nurse very trying. The interests of the nurse are best served by acting in such a way as to retain the sympathy of her patient. Under the above circumstances it would be most easily done by putting the position fairly before her and asking for half fees for the unoccupied fortnight.

I am very anxious to train as a maternity nurse, but I have not the money to pay the fees. Can you tell me if there is a fund from which I could obtain a loan for the purpose?—*Anxious One.*

There is no fund of the kind; but if you are eligible for training, why not take a three years' course at one of the infirmaries which include the midwifery course, as for instance, at Lewisham Infirmary? For such institutions refer to "The Nursing Profession: How and Where to Train."

*Laundry.*

(69) In presenting my account at the close of a month's maternity nursing my patient objected to pay the sum of 10s. for laundry as she thought it excessive. I informed her that half a crown a week was the usual charge, and she referred me to her husband. Some days later when I obtained an interview, he told me that "he left all domestic matters to his wife." My late patient then offered me 5s., and afterwards 6s., in settlement of my account, which offer I refused. Can I get my money through the County Court?—*Perplexed.*

Probably you could; but is it worth while? Accept the 6s., and in future have your charge for laundry printed in your scale of charges in order that there may be no dispute.

*Abroad.*

(70) Will you kindly tell me if there is a good opening for a nursing home in America, and in what place I should start?—*Matron.*

It is quite impossible for us to say.

I wish to know if there are any appointments open to English nurses in Japan, in Government hospitals or otherwise.—*Cyonro.*

For nursing in Japan apply for information to the Lady Superintendent, Doshisha Mission Hospital's Nursing Corps, Kyoto, Japan; for appointments abroad in Government hospitals apply the Secretary, the Colonial Nursing Association, Imperial Institute, S.W.

*Supra-Renal Extract.*

(71) Will you kindly tell me if supra-renal extract is liable to produce a high temperature and tonsilitis when first begun?—*Addisons.*

This question belongs to therapeutics and not to nursing.

*Home.*

(72) Will you kindly tell me of a home either at the seaside or in the country where a girl who suffers from occasional fits of epilepsy could be received? She needs a change badly, and the fits render her ineligible for most convalescent homes.—*District Nurse.*

You are close to the National Society for Employment of Epileptics at Chalfont St. Peters. Write to the Secretary, 12 Buckingham Street, Strand, W.C.

*Hospital Screens.*

(73) Can you tell me what sort of wood is used for making hospital screens, and what would be the approximate cost of a five-leaf screen with fittings, such as is used in the London hospitals?—*Provincial.*

The favourite screens are made of bamboo frames and chintz hangings. Any dealer in hospital specialities will get you one for a trifling cost.

*Uniform.*

(74) Miss O. would be obliged if the Editor could give her any information as to the uniform worn by the nurses at King's College Hospital. She would like a picture of the indoor uniform, and pattern of the dress and full particulars to aid her in making the same.

Miss O. should write to the sister-matron of the hospital for these particulars.

*Register.*

(75) Will you kindly tell me if there is a register of nurses residing in English hospitals and infirmaries, and where I might write for it?—*Anxious Enquirer.*

The nearest approach to a register of nurses is Burdett's Official "Nursing Directory," published by the Scientific Press.

*Queen's Nurse.*

(76) I am an Australian-trained nurse, and wish to become a Queen's nurse; will you kindly tell me how to set about it?—*G. A. H.*

Apply to the General Superintendent, St. Katherine's Precincts, Regent's Park, N.W.

**Standard Books of Reference.**

**"The Nursing Profession: How and Where to Train." 2s. net; post free 2s. 4d.**

**"Burdett's Official Nursing Directory." 3s. net; post free, 3s. 4d.**

**"Burdett's Hospitals and Charities." 5s.**

**"Hospital Expenditure: The Commissariat." 2s. 6d.**

**"The Nurses' Dictionary of Medical Terms." Cloth, 2s.; leather, 2s. 6d. net.; post free, 2s. 8d.**

**"Burdett's Series of Nursing Text-Books." 1s. each.**

**"A Handbook for Nurses." (Illustrated). 5s.**

**"The Physiological Feeding of Infants." 1s.**

**"The Physiological Nursery Chart." 1s.; post free, 1s. 3d.**

**All these are published by the Scientific Press, Ltd., and may be obtained through any bookseller or direct from the publishers, 28 and 29 Southampton Street, London, W.C.**

### Travel Notes.

**By Our Travelling Correspondent.**

### CXIV.—A WINTER IN FLORENCE.

The season has again come round for what one of my correspondents calls "fireside travelling," and I purpose to take Florence this year as the centre of our musings. There was a time when I, too, had to be content with fireside travelling, and an immense amount of pleasure I gained from it, and, I think, laid up a copious store of pleasant knowledge about the countries I hoped some day to visit—knowledge not very deep or erudite, perhaps, but such as gave me immense help and delight when I saw the historic and romantic spots already known to my inner eye, if I may use the expression.

### Books to be Studied.

There are many that will help you greatly to understand something of Florence and the Florentines, and I would counsel you by no means to despise fiction. The volume of all others that I think leads you the most deeply into the very heart of Florentine life is "Pascarel," by Ouida. That gifted authoress has lived many years in Italy, and learnt to know her adopted country with an intimacy very unusual with foreigners. "Pascarel" makes the best guide-book and companion that I know for the dweller in the beautiful city of lilies. In Hare's "Cities of Central Italy" you will find a splendid condensed history of Florence, and everything of real importance to the visitor is mentioned there. I think it is a pity that he is so one-sided in his opinion concerning the great questions and convulsions that have agitated the latter-day Italian politics; but then, if he is partial, he sins in glorious company—we have but to remember Macaulay. It must be almost impossible to an earnest nature not to allow itself a strong bias in speaking of questions of burning interest.

Another novel which gives a perfect impression of mediæval Florence is "Romola." I am bound to say I find it a little heavy, but it is correct and very instructive; still it does not give you the Italian "feeling" like a perusal of "Pascarel."

W. D. Howells, that charming American writer, gives us "A Florentine Mosaic" in his volume called "Tuscan Cities." He opens his account with a delightful sketch of life in the Piazza Santa Maria Novella; his writing is of that intimate, lively, confidential kind, without one trace of prosiness, which invariably leaves one with a desire for more of it.

You will of course study Ruskin's "Mornings in Florence," a most invaluable work, and it gives you much in little. I tremble to confess the fearful heresy—that it bores me, but I read it piously all the same. With all his learning, his real knowledge of his subject, and his exquisite language, for me there is too much Ruskin and too little Italy. I prefer to read in humbler literature.

Mrs. Oliphant's "Makers of Florence" and Horner's "Walks in Florence" are both interesting and instructive, and for books dealing a little more exclusively with art there are Perkins' "Tuscan Sculptors," Jameson's "Sacred Art," and Lindsay's "Christian Art."

### Expenses of Life in Florence.

This may be reckoned from 3s. 4d. per day up to anything you like in the palatial hotels on the Lung Arno, and it is a place where you can take a modest single room from 30 lire a month and add the luxury of attendance for another 5 lire. This is a very good plan for those who are out much and do not mind dining at a restaurant or having their dinner sent in from a trattoria.

Pensions abound of all kinds and degrees, and there are several very moderate native hotels. Cabs are cheap, 80c. the course, and 1 lire 30c. by the half hour, less after the first. Outside the walls naturally dearer—first half hour, 2 lire; second, 1 lire.

Trams and omnibuses make getting about very easy now, there is even an electric tram to grey old Fiesole, and also another to the celebrated Certosa.

### The Charm of Florence.

In what does this consist? It is a question almost impossible to answer, there is something so subtle in the spell which it casts over us, that words fail to analyse it. The feeling it evokes is quite different to that which gathers round the thought of Rome, though not less powerful. It is a tenderer sentiment that we give to Florence; partly, perhaps, because though it has been the scene of party strife, religious fanaticism, and cruelties, both public and private, it is not on the whole so stained with the atrocities caused by ambition, hatred, lust, and bigotry, as the Eternal City. This may be considered a far-fetched idea—very probably it is—but some such thought has crossed my mind as I compared the two places.

Then Florence is so bright and sunny, and the Arno is a less sullen river than the Tiber, and the life of the Tuscan populace is so many-sided and friendly, whilst the close resemblance, and in many respects unchanged aspects of existence in mediæval and present-day Florence give it a witchery for us unknown elsewhere. Rogers apparently had this feeling when he wrote these lines:—

"Of all the fairest cities of the earth,
None is so fair as Florence. 'Tis a gem
Of purest ray; and what a light broke forth,
When it emerged from darkness! Search within,
Without; all is enchantment! 'Tis the Past
Contending with the present; and in turn
Each has the mastery."

And then its recollections! Can one walk on the Lung Arno past the foot of the Ponte Vecchio where once the statue of Mars stood, and not see flowing there the warm young blood of Buondelmonte, flowing in expiation of a broken love troth or cross the bridge itself and not see in the mind's eye that roystering silversmith and artist Benvenuto Cellini, swaggering forth on some errand of vengeance or lawless love—or can we visit the Duomo and not hear again the burning words of Savanarola—or witness the treacherous murder by the Pazzi conspirators of Guiliano Medici, as the Host was elevated at High Mass—or again walk the quiet cloisters of San Marco, and not live over again the terrible day when a fight took place in the church—the beginning of the end—that tragic end by halter and fire in the Piazza Signoria. Can we look at the great buildings of the Medici and not remember that lonely and conscience-stricken death-bed where Il Magnifico was refused absolution, because even in death he would not relax his grip on Florentine liberty.

Of some of these interesting spots (with my readers' permission) and of their past history and present interest I hope to speak in the coming dark days of winter, when one's thoughts revert very pleasantly to sunny lands.

### TRAVEL NOTES AND QUERIES.

Bruges (All things to those who can wait).—Many thanks for your kind letter. I know the place very well, and it is all you describe. I always value letters of personal experience.

